# Supplementary material for: Metformin induces lipogenic differentiation in myofibroblasts to reverse lung fibrosis
Source: Nat Commun. 2019 Jul 5;10:2987. doi: 10.1038/s41467-019-10839-0 (PMC6611870; doi:10.1038/s41467-019-10839-0)
Supplement: Supplementary file 1 — Supplementary information [file 41467_2019_10839_MOESM1_ESM.pdf]

Supplementary Information

Metformin induces lipogenic differentiation in myofibroblasts to reverse lung fibrosis

Kheirollahi et al.

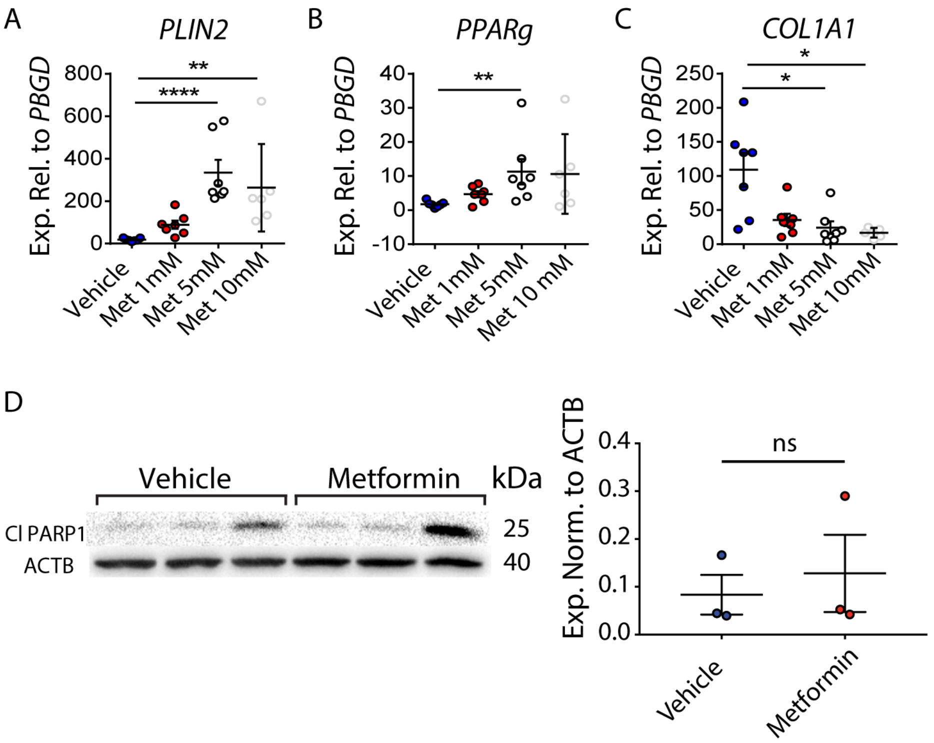

Supplementary Figure 1. Dose-finding study for treating human IPF lung fibroblasts with metformin. (A-C) qPCR analysis of *PLIN2, PPARg* and *COL1A1* in IPF fibroblasts treated with 1 mM, 5 mM or 10 mM metformin or vehicle. **(D)** Western blot showing the expression of the apoptotic marker cleaved PARP1 in fibroblasts treated with vehicle or 5 mM metformin. Quantification of the immunoblot is shown in the right panel. Each data point corresponds to one patient and error bars indicate s.e.m. (A-C) n=6-7 per group, (D): n=3 per group. Kruskal-Wallis test was used in (A-C) and Mann-Whitney test was used in (D). * $P<0.05$, ** $P<0.01$, **** $P<0.0001$. ns: Not significant.

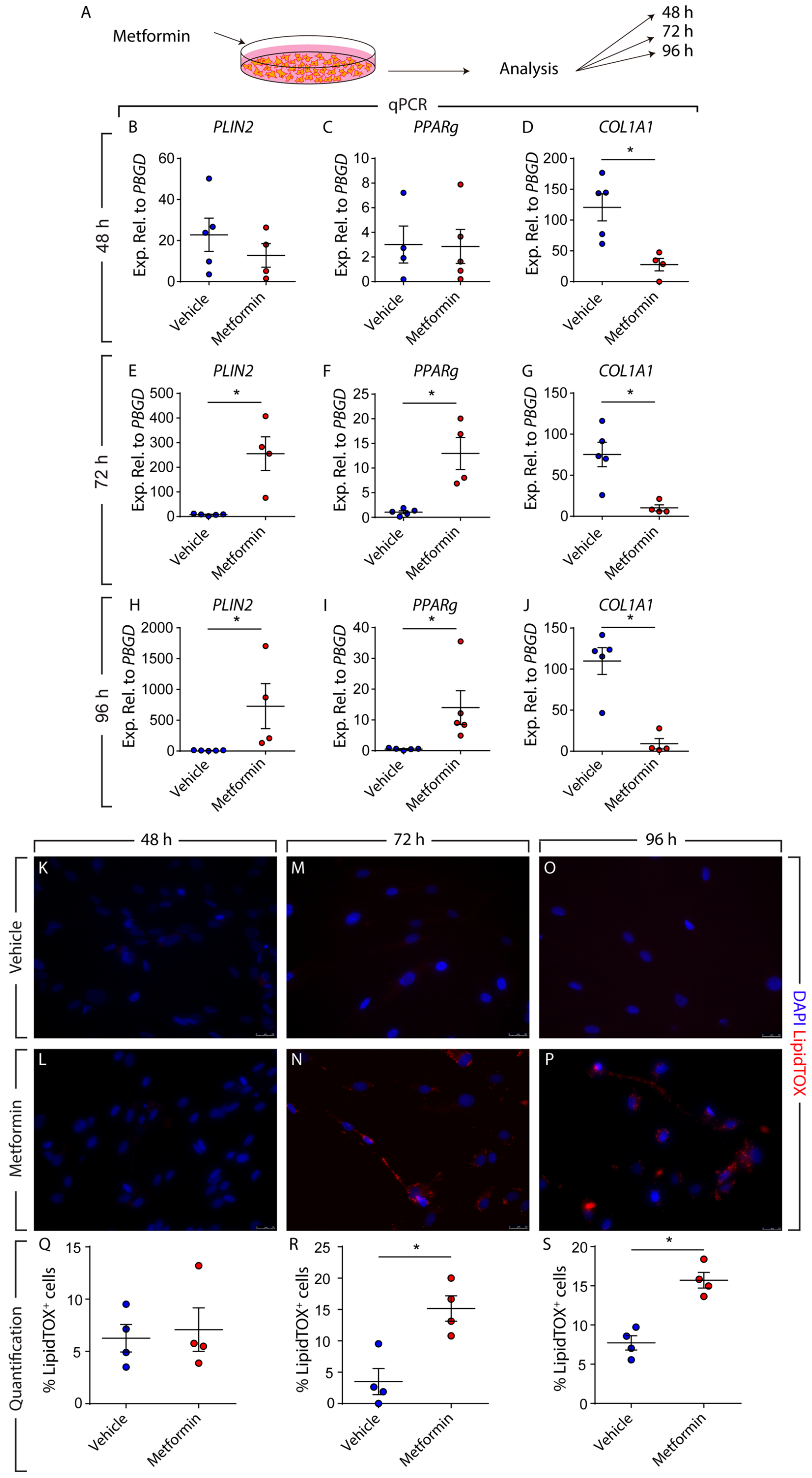

Supplementary Figure 2. Metformin induces lipogenic differentiation in IPF fibroblasts starting at 72 h after treatment. (A) Schematic representation of the experimental setup. **(B-D)** qPCR analysis of *PLIN2, PPARg* and *COL1A1* in IPF fibroblasts treated with 5 mM metformin for 48 h. A Similar analysis is shown after 72 **(E-G)** and 96 h **(H-J). (K-P)** Staining of metformin- and vehicle-treated cells with LipidTOX (red) and DAPI (blue) at 48, 72 and 96 h. **(Q-S)** Quantification of lipid-droplet accumulation is shown in (K-P). Scale bars: (K-P) 25 µm. Each data point corresponds to one patient and error bars indicate s.e.m. (B-J) n=4-5 per group. (Q-S) n=4 per group. Mann-Whitney test was used in (B-J, Q-S). * $P<0.05$.

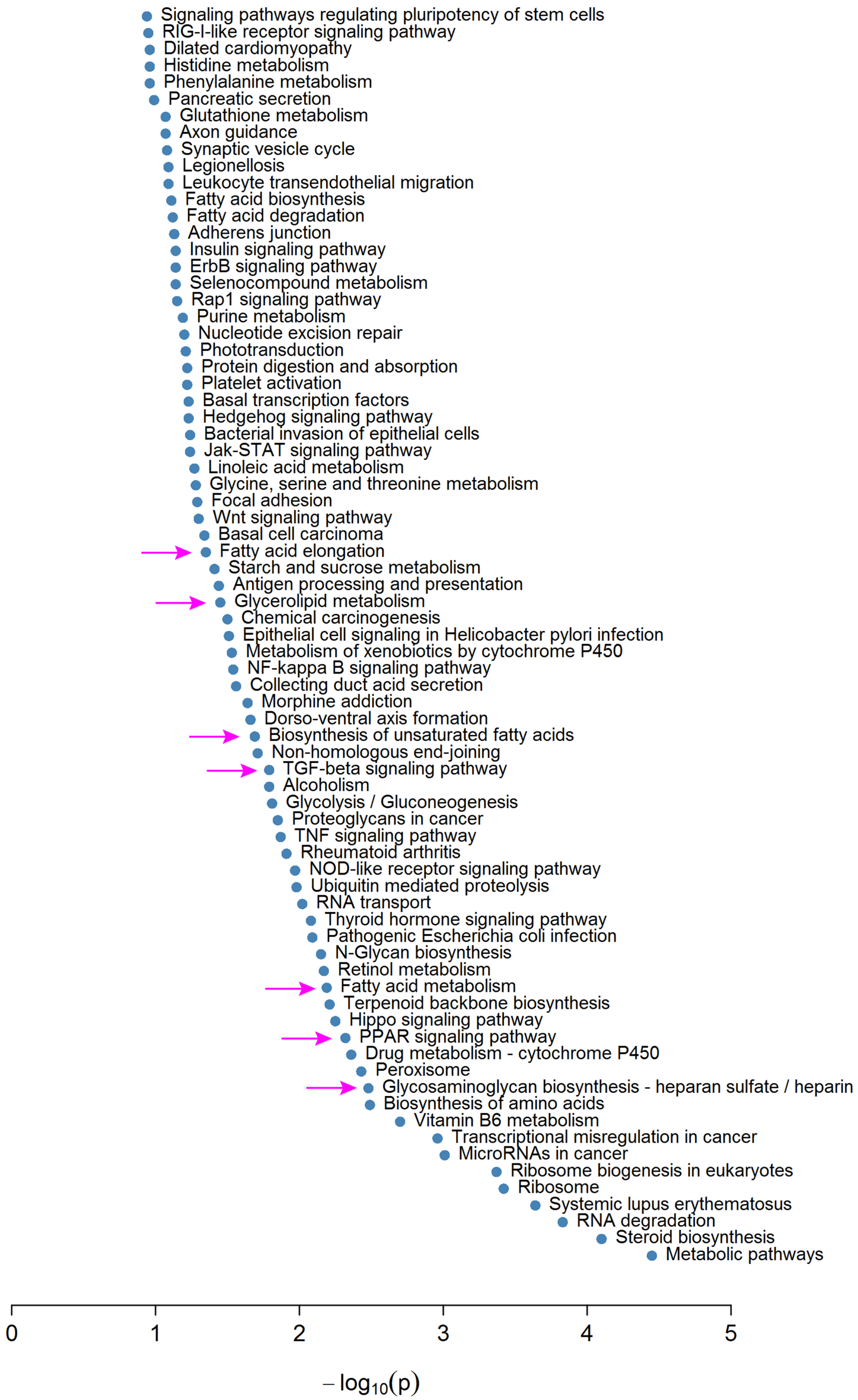

Supplementary Figure 3. KEGG significance plot showing signaling pathway alterations in metformin- and vehicle-treated human IPF lung fibroblasts. The top 75 candidate pathways are shown. Selected pathways are marked by the arrows. Vehicle-treated group: n=4, metformin-treated group: n=3.

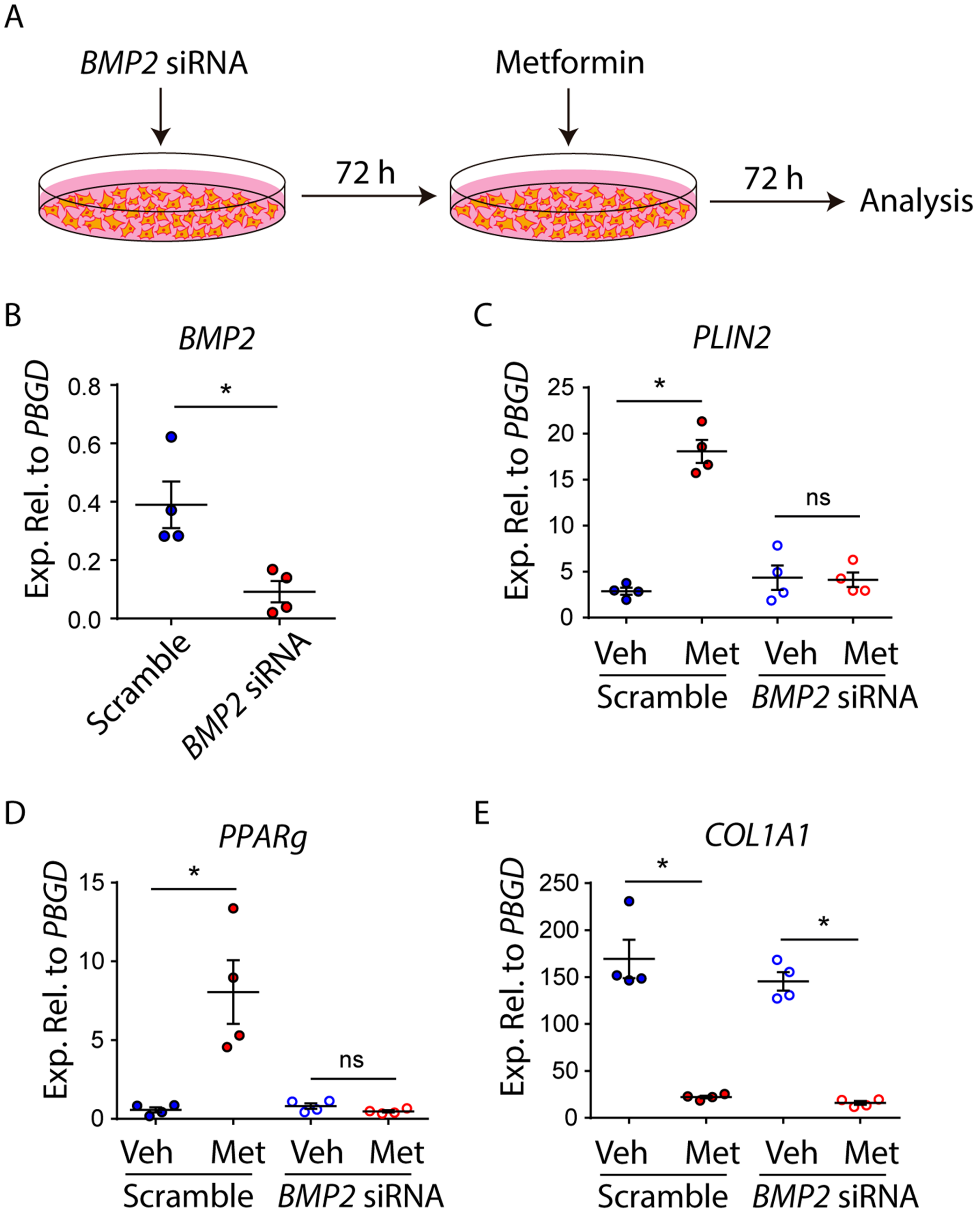

Supplementary Figure 4. *BMP2* knockdown inhibits metformin-induced lipogenic differentiation in IPF lung fibroblasts. (A) Schematic representation of the experimental setup. **(B-E)** qPCR analysis of *BMP2, PLIN2, PPARg* and *COL1A1* in human IPF lung fibroblasts treated with metformin in the presence of scramble or *BMP2* siRNA. Each data point corresponds to one patient and error bars indicate s.e.m. n=4 per group. Mann-Whitney test was used in (B) and Kruskal-Wallis test was used in (C-E). * $P<0.05$. Met: Metformin, ns: Not significant, Veh: Vehicle.

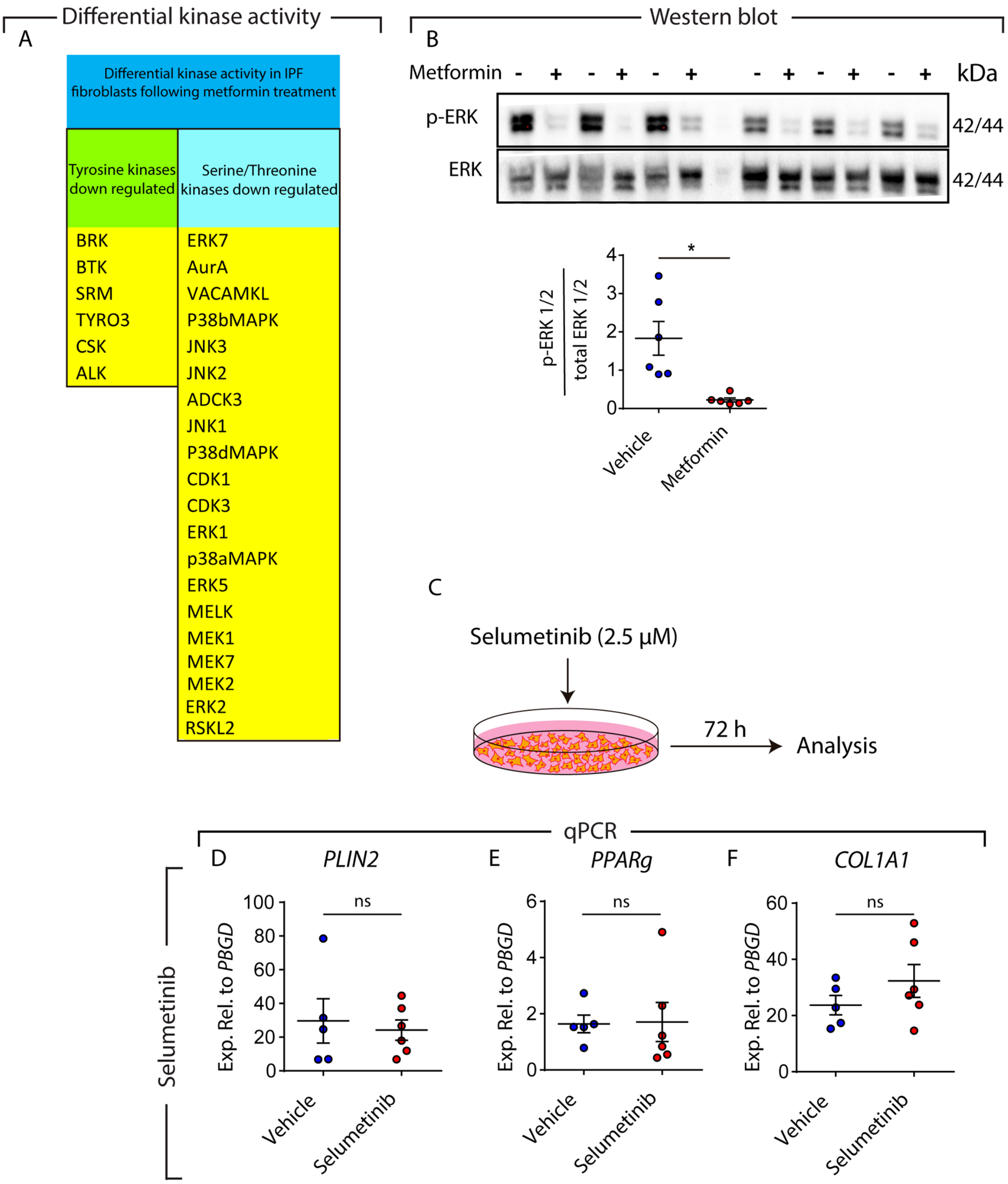

Supplementary Figure 5. Inhibition of ERK phosphorylation does not mediate the antifibrotic effect of metformin in human IPF lung fibroblasts. (A) Kinase activity assay as determined by PamStation analysis. **(B)** Western blot showing decreased p-ERK levels in response to metformin treatment. Quantification of p-ERK levels is shown in the lower panel. **(C)** Schematic representation of the experimental setup. **(D-F)** qPCR analysis of *PLIN2, PPARg* and *COL1A1* in IPF fibroblasts treated with selumetinib or vehicle for 72 h. Each data point corresponds to one patient and error bars indicate s.e.m. (B) n=6 per group. (D-F) Vehicle-treated group: n=5, Selumetinib-treated group: n=6. Mann-Whitney test was used. * $P<0.05$. ns: Not significant.

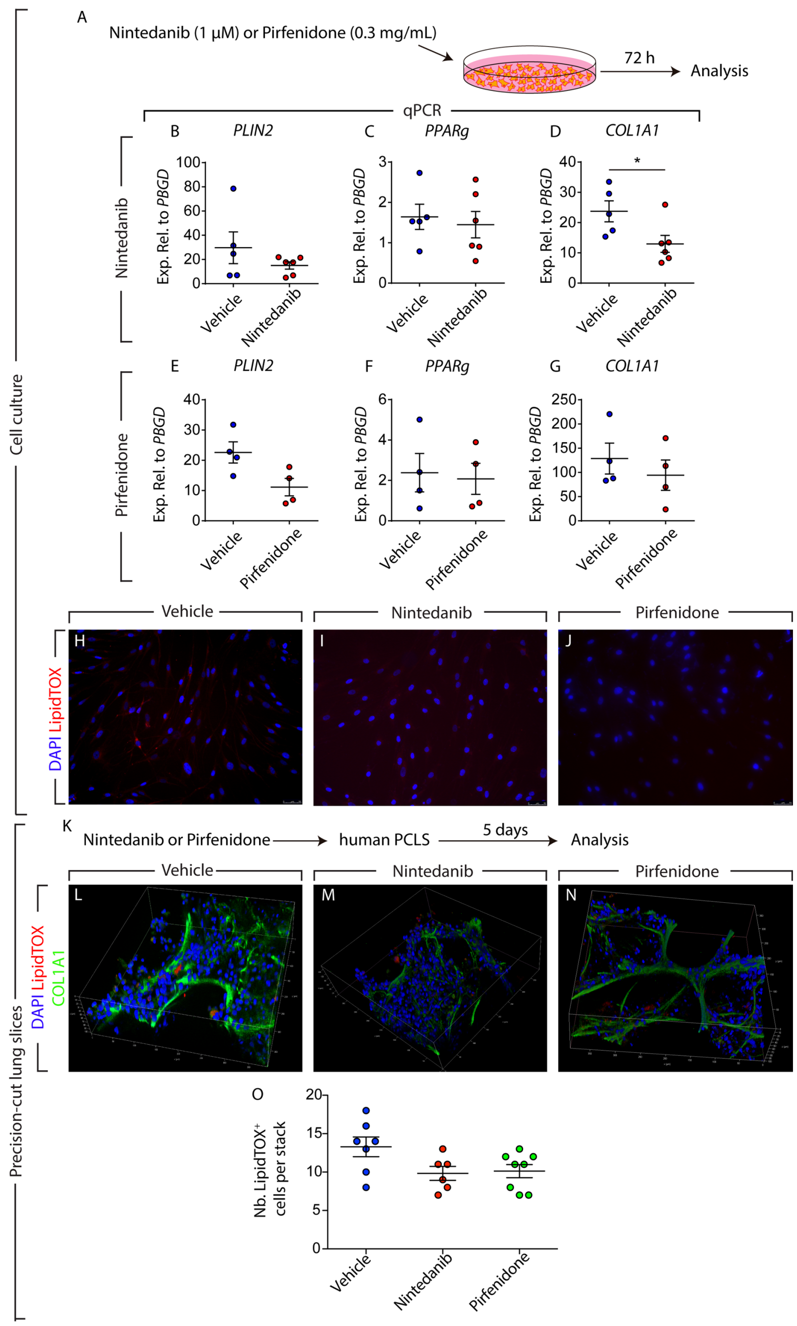

Supplementary Figure 6. Nintedanib and pirfenidone do not induce lipogenic differentiation in IPF lung fibroblasts. (A) Schematic representation of the experimental setup. **(B-D)** qPCR analysis of *PLIN2, PPARg* and *COL1A1* in human IPF lung fibroblasts treated with nintedanib. A similar analysis for pirfenidone-treated cells is shown in **(E-G). (H-J)** Staining of nintedanib-, pirfenidone- and vehicle-treated cells with LipidTOX (red) and DAPI (blue). **(K)** Schematic representation of the experimental setup using precision-cut lung slices (PCLS). **(L-N)** 3D-reconstruction of z-stacks of nintedanib-, pirfenidone- and vehicle-treated PCLS stained for COL1A1 (green) and lipid droplets (red). **(O)** Quantification of LipidTOX$^+$ cells as determined by manual counting across z-stacks. Scale bars: (H-J) 50 μm. (B-G) Each data point within a given group corresponds to one patient. (B-D) Vehicle-treated group: n=5, nintedanib-treated group: n=6. (E-G) n=4 per group. (O) Each data point within a given group corresponds to one z-stack and error bars indicate s.e.m. Vehicle-treated group: n=7, nintedanib-treated group: n=6, pirfenidone-treated group: n=8. Mann-Whitney test was used in (B-G) and Kruskal-Wallis test was used in (O). * P<0.05.
