# Supplementary material for: Metformin induces lipogenic differentiation in myofibroblasts to reverse lung fibrosis
Source: Nat Commun. 2019 Jul 5;10:2987. doi: 10.1038/s41467-019-10839-0 (PMC6611870; doi:10.1038/s41467-019-10839-0)
Supplement: Supplementary file 5 — Related Manuscript File [file 41467_2019_10839_MOESM5_ESM.pdf]

Related to Figure 5. *AMPK* knockdown using siRNA.

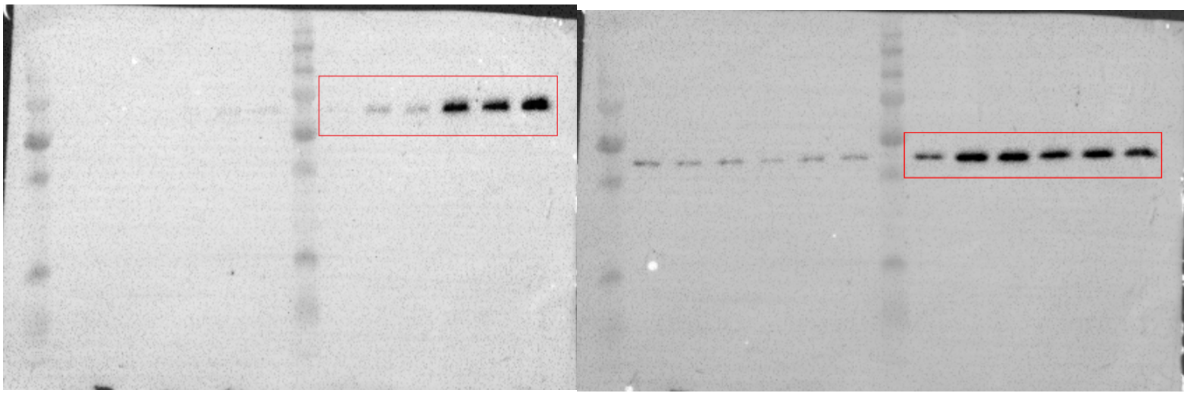

WB: AMPK (63 kDa)

WB: ACTB (42 kDa)

Related to Figure 6. *PPARg* knockdown using siRNA

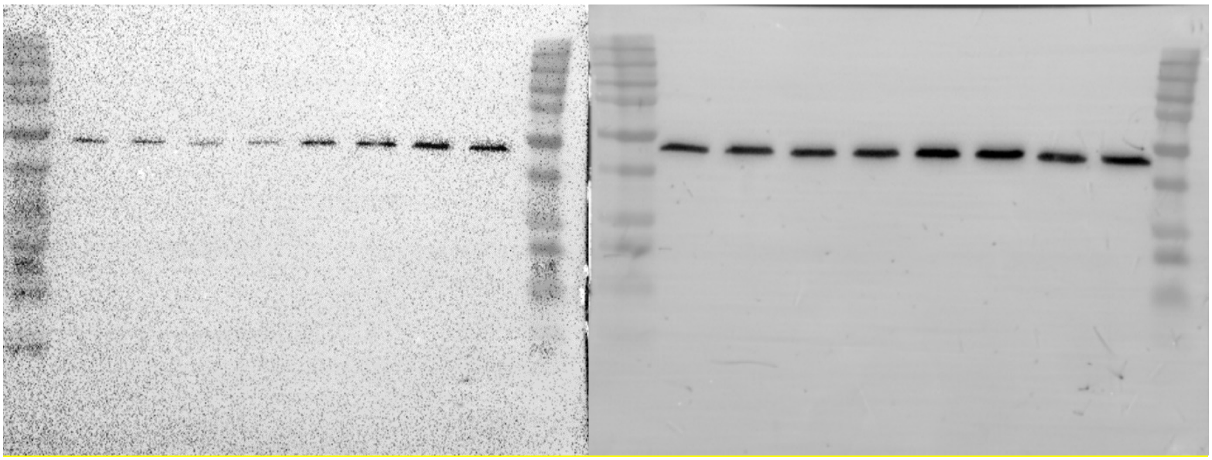

WB: PPARg (~50 kDa)

WB: ACTB (42 kDa)

Related to Figure 7. Treatment of human lung fibroblasts with metformin

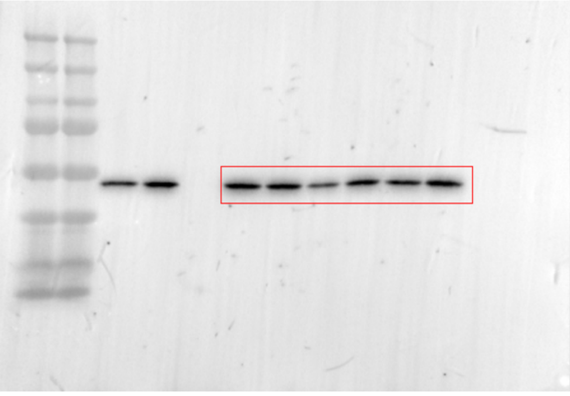

WB: PPARg (~50kDa)

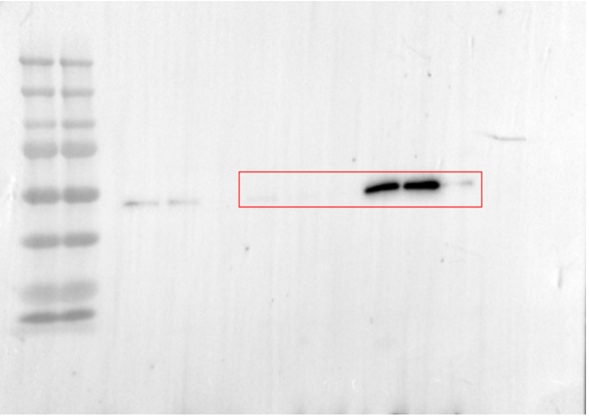

WB: p-PPARg (~50kDa)

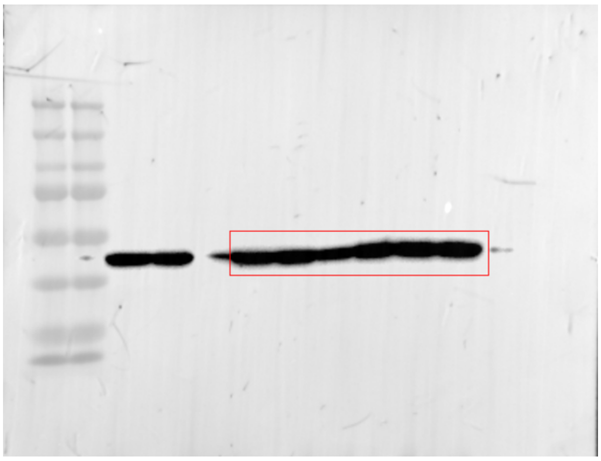

WB: ACTB (42 kDa)

Related to Figure 7. Phosphorylation of SMAD1/5/8

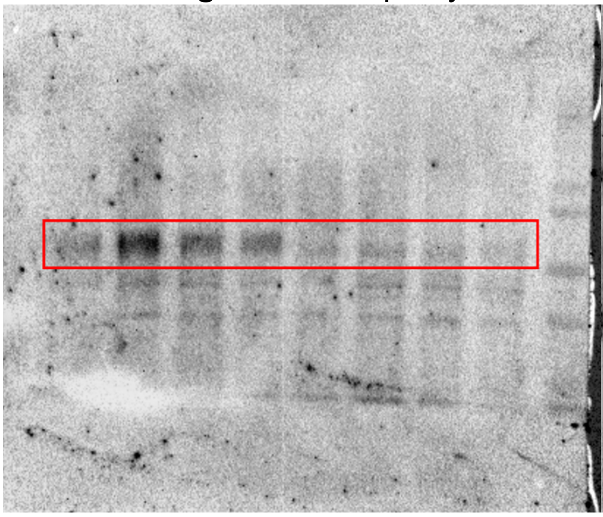

WB: p-SMAD1/5/8 (~60kDa)

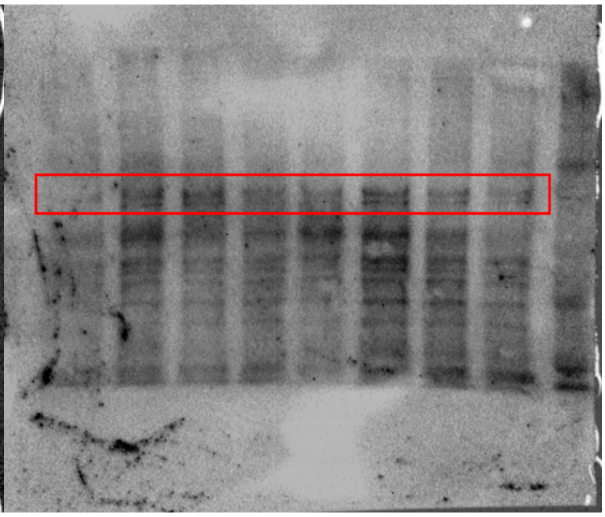

WB: Total SMAD1/5/8 (~60kDa)

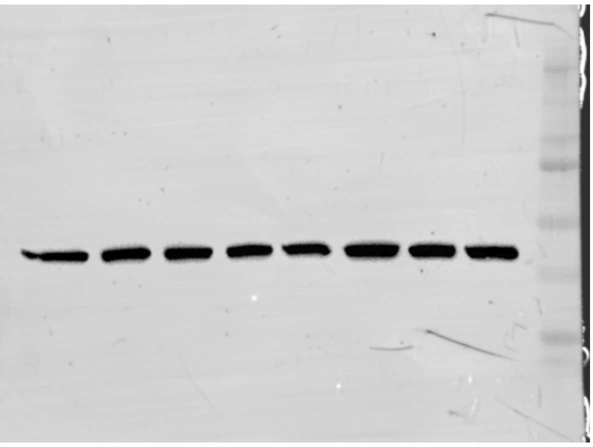

WB: ACTB (42 kDa)

Related to Figure S1.

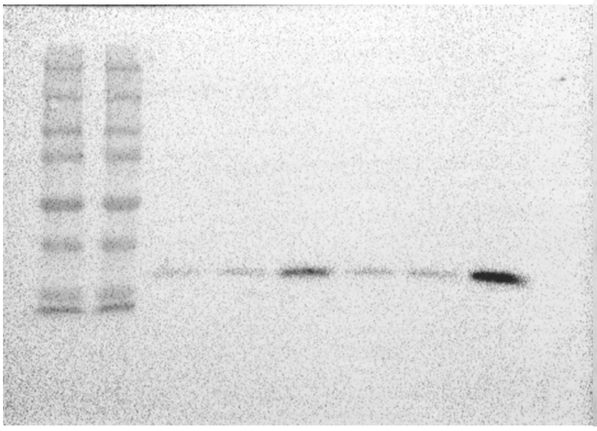

WB: cleaved PARP1 (~25kDa)

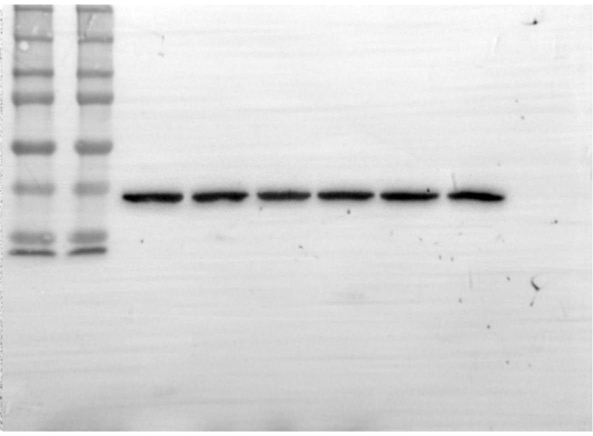

WB: ACTB (42 kDa)

Related to Figure S5.

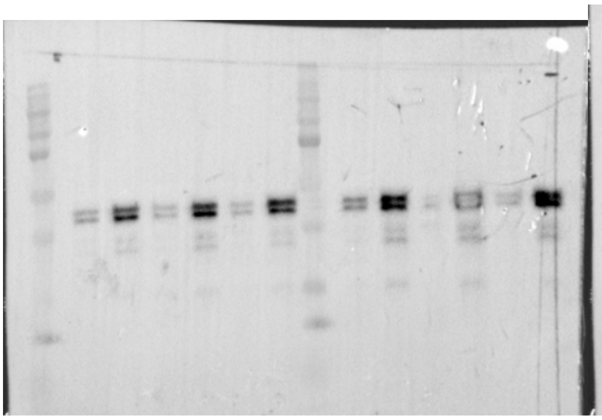

WB: p-ERK1/2 (40 kDa)

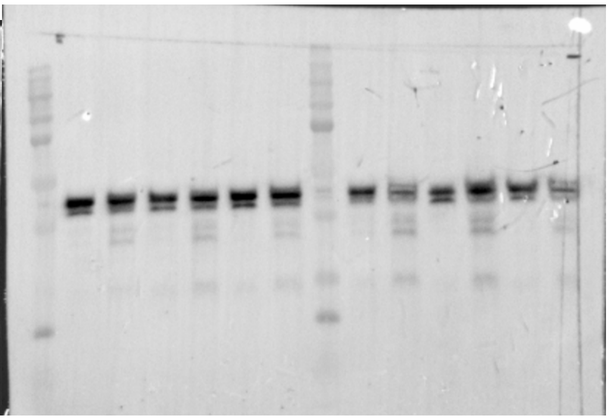

WB: Total ERK1/2 (40 kDa)
