# Supplementary material for: Metformin induces lipogenic differentiation in myofibroblasts to reverse lung fibrosis
Source: Nat Commun. 2019 Jul 5;10:2987. doi: 10.1038/s41467-019-10839-0 (PMC6611870; doi:10.1038/s41467-019-10839-0)
Supplement: Supplementary file 7 — Description of Additional Supplementary Files [file 41467_2019_10839_MOESM7_ESM.pdf]

Legends for supplementary movies

Supplementary Movie 1. Time-lapse imaging of vehicle-treated human IPF lung fibroblasts for 68 h. Brightfield and fluorescent images (for red LipidTOX stain) were acquired every 1 h. Scale bar: 100 μm.

Supplementary Movie 2. Time-lapse imaging of rhTGFβ1-treated human IPF lung fibroblasts for 68 h. Brightfield and fluorescent images (for red LipidTOX stain) were acquired every 1 h. Scale bar: 100 μm.

Supplementary Movie 3. Time-lapse imaging of metformin-treated human IPF lung fibroblasts for 68 h. Brightfield and fluorescent images (for red LipidTOX stain) were acquired every 1 h. Scale bar: 100 μm.
